# Providence Asylum for the Insane

**Published:** 1886-06

**Authors:** 


### Providence Asylum for the Insane and Inebriate.

In a former number of this journal, we spoke of the change in the management and medical staff of this most excellent institution. Since this time the facilities for receiving into the institution have been increased, another ward having been fitted up for women patients. In a short time there will be another men's ward added to the present number. Thus, with the growth of the institution, additional medical supervision was needed. To meet this want, two assistant physicians have been appointed—Dr. George W. T. Lewis, a graduate of Niagara University, and Dr. William G. Ring, of this city. The attending physicians, Drs. William Ring and Floyd S. Crego, have decided to attend the institution alternately, each serving three months. Dr. Lewis will assist Dr. Crego, and Dr. Ring, Jr., will assist Dr. Ring. In this manner the institution will be visited every day by one of the doctors on duty at that time.

In addition to the care of the insane, there is one ward devoted to the care of the inebriate who come voluntarity to the institution.

